# Editorial: Methods and protocols in nanotoxicology: volume II

**DOI:** 10.3389/ftox.2024.1532023

**Published:** 2025-01-03

**Authors:** Seyed Ali Johari, Il Je Yu, Eugenia Valsami-Jones

**Affiliations:** ^1^ Department of Fisheries, Faculty of Natural Resources, University of Kurdistan, Sanandaj, Kurdistan, Iran; ^2^ HCT Seattle, Seattle, WA, United States; ^3^ Department of Environmental and Occupational Health Sciences, University of Washington, Seattle, WA, United States; ^4^ School of Geography, Earth and Environmental Sciences, College of Life and Environmental Sciences, University of Birmingham, Birmingham, United Kingdom

**Keywords:** nanotoxicology, standard methods, fish, human, environment, nanomaterials

There is currently a major drive towards developing new applications in nanotechnology, including advanced material technologies. As a result, many engineered nanomaterials (ENMs) are coming along, promising new applications ranging from medicine and the food industry to agriculture and environmental remediation. However, alongside the innovation process for new materials, an urgent need exists to understand in precise terms the possible toxicity upon exposure that such materials might carry. This *Frontiers in Toxicology Research Topic*, entitled “Methods and Protocols in Nanotoxicology: Volume II,” pinpoints key contributions from four innovative articles that come together to develop a path leading to better methodologies in assessing the toxicological profiles of nanomaterials.

First, the article “Dechorionated zebrafish embryos improve evaluation of nanotoxicity” by Kim et al. draws on the limitation of the conventional model of zebrafish embryos due to their surrounding chorion, which restricts the permeability of nanomaterials. A very informative investigation with confocal and scanning electron microscopy techniques provided evidence that dechorionated embryos are much more sensitive to nanomaterials than their chorion-intact peers, based on lower LC_50_ values for several ENMs. Notably, this work is calling for refinement in animal testing practices according to the “3Rs” approach but has also proposed a standardized technique, ISO/TS 22082:2020, which might significantly improve nanotoxicity assessment (ISO, 2020).

The second contribution, “*In vitro* micronucleus assay: Method for assessment of nanomaterials using cytochalasin B” by Farabaugh et al., proposes an advance in the *in vitro* micronucleus assay by detailing a protocol for testing the aneugenic and clastogenic potentials of nanomaterials. This robust methodological approach overcomes the principal challenges in testing nanomaterials by introducing strict criteria for selecting cell lines, dose determination, and exposure conditions. Most importantly, cytochalasin B will ensure that the cells being analyzed represent cell division, giving more accurate quantitative data on DNA damage and micronuclei formation. The authors present this important assay in a step-by-step fashion to make it available for researchers in nanotoxicology.

In the third article, “Investigating nanoplastics toxicity using advanced stem cell-based intestinal and lung *in vitro* models” by Busch et al. tackles the alarming rise of nanoplastics in the environment and identifies the pressing need to check the health effects of such exposure. The authors proposed the use of a stem cell-based technique over conventional alternatives based on cancer cell lines since it is far closer to real human physiology. By applying advanced exposure techniques, the authors demonstrate how such models can deliver valuable data on the mode of action of nanoplastics, giving new insights into their health consequences. The comparison executed here focuses on benefits derived from stem cell applications and some of the current methodological bottlenecks in nanotoxicology.

Finally, the article “Protocols for isolation and characterization of nanoparticle biomolecular corona complexes” by Soliman et al. covers protocols addressing nanoparticle-protein corona (NP-PC) formation in detail. The authors propose that biological fate or outcome, such as toxicity, uptake and biodistribution, is predetermined by the interaction of engineered nanoparticles with biological molecules found in the surrounding media. Several isolation and characterization techniques targeting biomolecular corona complexes are described by the authors, including magnetic isolation methods for superparamagnetic nanoparticles and advanced physicochemical characterization approaches. By explaining these protocols, the authors underline the importance of examining the biomolecular corona to predict more reliably potential hazards from engineered nanoparticles.

These four articles together constitute a valuable resource for the nanotoxicology community while providing methodology approaches and strengthening the use of innovative models and techniques for improving the understanding of the interaction of nanomaterials in biological systems. The combined results sketch a scenario wherein the future of nanotoxicology is in integrating diverse experimental methodologies relevant to human biology and exposure conditions.

In this intriguing area where nanotechnology and toxicology overlap, many opportunities and challenges remain to refine and develop new protocols that answer ethical research and pressing public health concerns. The scientific community’s commitment to new methodologies and encouragement of collaborative research will be significant in the safe and responsible advancement of nanotechnology. We hope this Research Topic will catalyze future research efforts toward uncovering the complex interactions between engineered nanomaterials and their related health effects.

We want to thank the authors for their valuable contributions to this volume and also the reviewers for their comments, which have greatly improved the quality of these works. We hope that this will stimulate further discussion in the field of nanotoxicology and inspire researchers to go beyond the existing techniques and to develop the real potential of nanomaterials in a safe and responsible way.
